# Comparing emergency medical system governance in Japan and South Korea: lessons for high-income countries from a multisource comparative health systems analysis

**DOI:** 10.12701/jyms.2026.43.3

**Published:** 2025-12-18

**Authors:** Kentaro Kajino, Jung Ho Kim, Jeong Ho Park, Kyoung-Jun Song, Mohamud R. Daya, Yasuyuki Kuwagata

**Affiliations:** 1 Department of Emergency and Critical Care Medicine, Kansai Medical University, Hirakata, Japan; 2 Department of Emergency Medicine, Yeungnam University College of Medicine, Daegu, South Korea; 3 Department of Emergency Medicine, Seoul National University Hospital, Seoul National University College of Medicine, Seoul, Korea; 4 Department of Emergency Medicine, SMG-SNU Boramae Medical Center, Seoul, Korea; 5 Department of Emergency Medicine, Oregon Health & Science University, Portland, OR, USA

**Keywords:** Emergency medical services, Health policy, Health services administration, Japan, Republic of Korea

## Abstract

**Background:**

Japan and South Korea, two advanced East Asian nations with universal health coverage and similar demographic challenges, have developed markedly different emergency medical services (EMS) systems. Despite growing interest in international benchmarking, structured, comparative studies that yield policy-relevant insights remain limited.

**Methods:**

We conducted a multisource comparative health-systems analysis using statutory laws, government publications, academic society reports, peer-reviewed literature, and national statistics. Key domains included EMS governance, workforce, prehospital organization, hospital-based emergency care, legal obligations for EMS patient transport and hospital acceptance, and governance and quality assurance mechanisms. Data were synthesized in comparative tables and narrative summaries to highlight structural and operational differences.

**Results:**

Japan’s EMS system operates under decentralized municipal control through 722 fire departments, serving 4,100 designated emergency institutions with 6,139 board-certified emergency physicians. In 2023, over 6.64 million ambulance dispatches occurred, and 8.6% were classified as critical cases (1.3% death and 7.3% severe). Korea’s EMS system is centrally governed with 412 designated facilities in a tiered system and 2,464 specialists. Annual ambulance activations exceeded 3.5 million, with severe cases accounting for approximately 5% to 10%. Japan employs a multilayered legal–institutional structure, primarily involving the Fire Service Act and the Medical Practitioners Act, allowing clinical discretion, whereas Korea enforces unified regulations with stricter obligations and criminal penalties for hospital refusal of emergency patients.

**Conclusion:**

The contrasting systems suggest that hybrid governance that combines centralized standard settings with local operational flexibility may optimize EMS performance. These findings provide lessons for EMS reform, cross-border collaboration, and disaster preparedness in high-income nations facing similar demographic and healthcare challenges.

## Introduction

Emergency medical services (EMS) systems serve as the foundational basis for a country’s response to time-sensitive emergencies such as out-of-hospital cardiac arrest (OHCA), severe trauma, and mass-casualty incidents. Although many nations have developed sophisticated EMS frameworks, their philosophies, governance, and operational protocols differ widely due to historical, legal, and sociopolitical factors [1,2].

Japan and South Korea, two neighboring East Asian countries, share similar socioeconomic development, healthcare infrastructures, and population health challenges. Both operate universal health insurance programs and provide nationwide 119 EMS access while facing mounting pressure from an aging population and an uneven distribution of health professionals. However, the EMS systems in Japan and South Korea differ substantially in terms of centralization, legal authority, physician roles in prehospital care, and medical oversight [1,3].

Understanding these differences is vital, as international collaboration in emergency medicine increases through registry-based studies and cross-border training efforts. Comparative analysis can reveal the strengths and limitations of each system, enabling mutual learning and system improvements. Such examples provide valuable reference points for EMS reform across Asia and beyond.

In recent years, large-scale multinational registries such as the Pan-Asian Resuscitation Outcomes Study and Pan-Asian Trauma Outcomes Study have contributed significantly to the advancement of EMS research across Asia [4]. In some cases, the general features of each country’s EMS system have been introduced in the context of specific clinical outcomes, such as survival after OHCA or trauma-related mortality and morbidity [4].

Despite the growing recognition of the importance of such comparative research, few studies have offered a structured two-country evaluation of the EMS systems in Japan and South Korea. Much of the existing literature consists of single-country reports or broad regional summaries that do not fully address the institutional, legal, or operational distinctions [1,2]. A more detailed examination is needed to better understand how these systems differ and what lessons may be drawn from their evolution and current status.

This study addresses this gap by conducting a comparative review of the EMS systems in Japan and South Korea. The goal is to document how each system has developed over time and how it currently functions, focusing on institutional design, regulatory context, and system-level organization.

## Methods

**Ethics statement:** This study analyzed publicly available aggregate data and did not require individual patient consent. The study protocol was reviewed and approved by the Institutional Review Board (IRB) of Kansai Medical University (IRB No. 2025265). All data handling and reporting adhered to the ethical guidelines for secondary data analysis.

### 1. Study design and approach

This study employed a targeted literature review and multisource documentary analysis to examine the structural, organizational, and legal dimensions of EMS in Japan and South Korea. We purposefully identified key documents that provided authoritative data on EMS governance frameworks by combining peer-reviewed literature, official government statistics, legal texts, and professional society reports.

### 2. Literature search and document selection

Documents were identified through multiple sources: (1) searches of English (PubMed, Scopus), Japanese (CiNii, J-STAGE, Ichushi Web), and Korean (KISS, KoreaMed, RISS) databases; (2) direct consultation of government agency websites (Fire and Disaster Management Agency [FDMA] of Japan; National Fire Agency [NFA] of South Korea; Ministry of Health, Labour and Welfare [MHLW] of Japan; Ministry of Health and Welfare [MOHW] of South Korea); (3) legal databases (e-Gov Laws and Regulations Search [Japan]; Korea Legislation Research Institute [KLRI]); and (4) professional society reports (Japanese Association for Acute Medicine [JAAM]; Japanese Medical Specialty Board [JMSB]; Korean Society of Emergency Medicine [KSEM]).

Database searches covered the period from January 2000 to July 2025, using keywords related to EMS, EMS governance, and health system structure in each language. No language restrictions were applied beyond English, Japanese, and Korean. Detailed search strategies and specific search terms for each database are provided in Supplementary Table 1.

Documents were selected based on their relevance to five thematic domains: (1) specialist training and certification, (2) prehospital EMS organization, (3) hospital-based emergency care structure, (4) legal obligations for patient transport and acceptance, and (5) governance and quality assurance mechanisms. The most recent and authoritative sources in each domain were prioritized. Peer-reviewed articles, government reports, white papers, legal documents, and official statistics published between 2000 and 2025 that provided system-level data or structural analyses were included. The exclusion criteria were clinical outcome studies without system-level analyses, opinion pieces without empirical data, and publications in languages other than English, Japanese, or Korean. Detailed inclusion and exclusion criteria are provided in Supplementary Table 2.

Following a systematic screening and eligibility assessment, 40 documents are directly cited in the manuscript. These include peer-reviewed articles (n=20), government reports, white papers (n=24), legal documents (n=10), professional society reports (n=8), and other authoritative sources (n=18). A simplified PRISMA (Preferred Reporting Items for Systematic Reviews and Meta-Analyses)-style document selection flowchart is provided in Supplementary Fig. 1.

### 3. Data extraction and analysis

Quantitative data on EMS structure, workforce, and operational performance were systematically extracted from official sources: FDMA annual reports and MHLW statistics for Japan, and NFA annual reports and MOHW emergency medical statistics for South Korea. Specialist certification data were obtained from the JAAM and KSEM.

Legal frameworks were analyzed using primary legislative texts retrieved from the e-Gov Laws and Regulations Search (Japan) and the KLRI databases. Key provisions governing hospital acceptance obligations, the EMS crews’ scope of practice, and medical oversight were translated and interpreted with input from coauthors with legal expertise.

The extracted data were organized using a thematic framework covering the five domains listed above. Comparative tables were developed to systematically compare the structural, workforce, legal, and operational characteristics of the two countries.

### 4. Validation process

To ensure validity and contextual accuracy, preliminary findings and data interpretations were reviewed and validated through iterative discussions among all coauthors (n=6), who collectively represented expertise across multiple domains relevant to this comparative analysis. The authorship team included (1) two emergency physicians with Medical Control (MC) physician certification from Japan, both with >20 years of clinical experience in tertiary emergency centers; (2) three emergency physicians and medical directors from South Korea, with direct experience in EMS medical oversight and hospital emergency department (ED) operations; and (3) one emergency physician and medical director from the United States, providing an international comparative perspective on EMS governance models.

All coauthors independently reviewed the draft comparative tables, quantitative data accuracy, cross-national interpretations, and legal framework analyses. Discrepancies in data interpretation and terminology were resolved through consensus discussions in coauthor meetings. This collaborative validation process ensured that the findings accurately reflected the operational realities and legal contexts of the EMS systems in both countries. Although formal inter-rater reliability calculations were not performed, the iterative review process across multiple domain experts with direct system knowledge provided a robust validation of the comparative findings.

### 5. Data triangulation and quality control

To enhance data reliability, we employed triangulation by cross-referencing multiple sources for key statistics. The emergency medicine specialist counts were verified across professional society membership databases (JAAM, KSEM) and government certification records. Ambulance transport volumes and operational metrics were cross-checked between fire agency reports (FDMA, NFA) and health ministry statistics (MHLW, MOHW). The legal provisions were confirmed through consultations with primary legal texts and secondary policy analyses. Where discrepancies were identified, the most recent and authoritative source was prioritized, and all data sources are explicitly cited in the comparative tables.

## Results

### 1. Specialist training and certification

The establishment of emergency medicine societies in Japan and South Korea was a policy response to the underdeveloped state of EMS systems during periods of rapid economic growth. The JAAM was founded in 1973 and initially focused on trauma care before expanding to include all aspects of emergency medicine [5]. Table 1 provides a comparative overview of the specialist systems in both countries. In contrast, the KSEM was established in 1989 with a clear orientation towards adopting the North American Emergency Medicine model [6]. Its founding purpose was to train “comprehensive emergency physicians” to manage all acute patients across internal medicine, surgery, pediatrics, and beyond. The society’s development was strongly influenced by exchanges with the American College of Emergency Physicians and the experiences of Korean physicians trained in the United States [7].

As of January 2025, Japan had 6,139 board-certified emergency medicine specialists accredited by the JAAM [8]. According to the JMSB, there are 108,430 certified specialists across 19 basic medical specialties [9], and emergency medicine accounts for approximately 5.7% of that pool. These figures place emergency medicine in the middle among Japan’s major specialties and show a steady upward trend, particularly in tertiary care medical centers. The number of emergency medicine specialists per 100,000 population is 4.96 [10]. In South Korea, approximately 2,464 emergency medicine specialists were certified by the KSEM as of 2024. According to MOHW statistics, this corresponds to approximately 2.5% of the national total of 97,186 specialists [11]. The number of emergency medicine specialists per 100,000 population is 4.81 [11,12]. Although the absolute number is lower in South Korea than in Japan, the relative proportion based on population size is similar.

In Japan, emergency medicine specialists are allowed to obtain additional board certifications in other traditional specialties such as internal medicine, surgery, and orthopedics. The JMSB does not prohibit multiple certifications, which allows physicians to develop broad cross-disciplinary clinical capabilities [13]. Subspecialty development in Japan is generally managed directly by related academic societies rather than by the JAAM. Additional certifications in fields such as trauma, critical care medicine, clinical toxicology, and burn injury are common. In South Korea, there is also no regulation prohibiting the acquisition of additional board certification after acquiring a specialist qualification. In fact, during the early days of emergency medicine in South Korea, many individuals who were already certified in specialties such as surgery or internal medicine went on to obtain board certifications in emergency medicine. However, owing to the lengthy training period and limited need for double-board certification, it is uncommon for Korean physicians to acquire more than one specialist qualification [14]. Moreover, in Korea, men are generally required to complete approximately 20 months of mandatory military service, whereas physicians are obligated to serve for an extended period of 36 months. Following board certification in emergency medicine, physicians may pursue additional training and academic recognition in subspecialties, such as toxicology, trauma surgery, critical care, pediatric emergency medicine, disaster medicine, and prehospital care. This is similar to the pathway followed by graduates of emergency medicine residency programs in the United States certified by the American Board of Emergency Medicine. These subspecialties are formally recognized by the KSEM and related societies through structured programs involving education, clinical practice, and assessments [7]. Although these are not national legal qualifications, they are considered authoritative designations within clinical institutions and are often prerequisites for appointments to leadership roles in specific departments, academic positions, or disaster response teams.

### 2. Prehospital emergency medical service organization

Japan and South Korea operate their EMS systems primarily through fire departments. Despite this structural similarity, the two countries exhibit fundamental differences in administrative frameworks, crew composition, scope of prehospital procedures, and command center operations. These differences are deeply rooted in national legislation, administrative culture, and healthcare delivery models. Table 2 provides a summary comparison of the EMS systems in Japan and South Korea.

In Japan, under the Fire Service Organization Act [15], EMS are decentralized and operated by individual municipalities. The FDMA provides national guidance and support but does not directly manage local operations. As of 2024, there were 722 fire departments across Japan, typically organized by municipalities or regional alliances. Each fire department operates its own communication command center, which handles 119 emergency calls, dispatches ambulances, and provides communication support. In recent years, regional integration has led to the formation of larger command centers [16,17]. In South Korea, EMS is centrally managed by the NFA under the Ministry of the Interior and Safety. This unified system was formalized in 2017. The system comprises one National Fire Headquarters and 19 Regional Fire Headquarters, aligned with each province or metropolitan city. In this scenario, there are approximately 250 fire stations and numerous 119 Safety Centers (comparable to substations). Each regional headquarters and fire station houses a 119 Integrated Command Center equipped with advanced information technology systems, enabling real-time dispatch coordination and support [18].

In Japan, each ambulance is typically staffed by three personnel, including at least one nationally licensed emergency lifesaving technician (ELST). ELSTs represent the *highest* level of prehospital providers in Japan and are authorized to perform defibrillation, advanced airway management, intravenous access, and epinephrine administration under online or offline physician medical direction. In addition to ELSTs, emergency medical technicians (EMTs) are general ambulance personnel who undergo over 250 hours of standardized emergency care training (including basic life support, trauma care, ambulance ride-along training, and equipment operation) after initial firefighting experience. Unlike ELSTs, EMTs provide basic prehospital care and assist ELSTs during emergency operations. Some ELSTs have completed additional accredited training, allowing them to perform designated advanced procedures under specific protocols. Medical direction is guided by local MC councils, ensuring physician oversight of all advanced interventions [19]. According to the latest statistics as of April 1, 2024, Japan has 5,415 emergency response teams; 6,640 ambulances (including reserve vehicles); 67,006 emergency medical personnel; and 33,350 certified ELSTs (EMS personnel with ELST qualifications) [17]. Currently, 99.6% of all emergency response teams have at least one certified ELST, demonstrating a high level of advanced life-support capabilities within the Japanese EMS system. All 722 fire departments in Japan operate EMS systems, providing coverage for virtually the entire population [17].

In South Korea, ambulance teams typically consist of two to three personnel, usually including at least one Level 1 EMT with a nationally certified qualification and broad prehospital capabilities. Level 1 EMTs are legally authorized to perform advanced procedures, such as defibrillation, airway management, intravenous access, and selected drug administration under the online physician’s medical direction. The scope of ELST practice in Japan is quite similar to that of Level 1 EMTs in South Korea; both scopes of practice look like that of advanced EMTs in the United States. In certain regions, emergency physicians are stationed at command centers to provide additional online medical oversight through radio or digital communication. Since 1996, licensed registered nurses employed as firefighter civil servants have been routinely assigned as crew members on 119 ambulances operated by South Korea’s fire departments [20]. As of December 2024, South Korea operated 1,881 ambulances (including reserve vehicles), employing 14,236 emergency medical personnel under the 119-ambulance service system. Of these individuals, 5,347 were certified as Level 1 EMTs, 2,253 were certified as Level 2 EMTs, and 4,290 held registered nurse licenses. These figures reflect ongoing efforts to enhance advanced prehospital care capabilities by integrating highly trained medical professionals into frontline EMS systems [21].

### 3. Hospital-based emergency care structure

Japan and South Korea have developed different frameworks for hospital-based emergency care systems, particularly in terms of how patients are triaged and distributed to appropriate medical facilities. In this section, the structural characteristics, operational principles, and societal contexts of each system are compared. Japan employs a nationally standardized, three-tier emergency care system that is stratified based on the severity of the patient’s condition. Primary Emergency Care is for mild cases (e.g., those who can visit clinics by themselves) and is typically provided at after-hours clinics, nighttime medical centers, and by on-call local physicians. Secondary Emergency Care addresses moderate cases requiring inpatient care and is provided by mid-sized hospitals designated as emergency medical institutions that operate on a rotation or full-time basis. Tertiary Emergency Care focuses on severe trauma, multisystem injuries, and cases requiring intensive treatment delivered at advanced facilities, such as tertiary care emergency centers and university hospitals with specialized teams and equipment [22]. In contrast, South Korea employs a tiered classification model for emergency medical facilities that focuses on institutional functions and regional healthcare capacity rather than strict patient severity. Local Emergency Medical Institutions provide initial care for minor emergencies, usually at small- to mid-sized hospitals. Local Emergency Medical Centers manage moderate-to-severe cases at typically larger general hospitals with higher diagnostic capabilities. Regional Emergency Medical Centers handle the most critical cases, including trauma and pediatric emergencies, across wide geographic areas, generally at designated university hospitals or national centers [23,24].

A notable distinction between the two countries lies in how they coordinate the transportation of emergency patients. In Japan, a well-established MC system enables paramedics to make hospital destination decisions based on protocols, past case data, and real-time consultations with online MC and receiving hospital physicians. Triage and destination selection are formalized, with patients allocated to institutions according to their conditions and available resources [22]. In South Korea, although EMTs are trained to assess severity, transport decisions are often made onsite by EMS crews or at the patient’s request. Especially in mild cases, patients frequently select their preferred hospital, leading to overcrowding at high-level centers. Thus, Japan’s system emphasizes centralized coordination and clinical appropriateness, whereas South Korea’s system tends to prioritize field-based flexibility and patient autonomy [25,26]. A summary of these comparisons is presented in Table 3.

The structure and operational scale of emergency medical institutions in Japan and South Korea reflect the distinct characteristics of each country’s healthcare system and emergency care policies. This section compares the number of emergency medical facilities, annual ED visits, distribution of patient severity, and workforce composition based on the most recent public statistics and official reports (2022–2024). In Japan, approximately 4,100 facilities are designated as emergency medical institutions, ranging from primary to tertiary care [27]. Among these, approximately 300 are tertiary Critical Care Medical Centers [28] and approximately 3,500 facilities provide secondary-level emergency care [27]. According to the FDMA, approximately 6.64 million patients were transported by EMS ambulances in 2023. Of these, 1.3% and 7.3% were mortality and severe cases, respectively (totaling 8.6% critical cases), 42.9% were moderate (requiring hospitalization), and 48.5% were mild (outpatient care) [17]. By contrast, approximately 412 facilities in South Korea are designated as emergency medical institutions by the MOHW, comprising 42 regional emergency medical centers, 135 local emergency centers, and 235 local emergency institutions [29]. National statistics indicate that severe cases (requiring intensive care unit stay, surgery, or hospital admission) account for 5% to 10%, moderate cases for 20% to 25%, and mild cases for approximately 70% of all ED presentations [30]. Japan employs a decentralized approach with many designated institutions, allowing broad access to emergency care. Conversely, South Korea utilizes a centralized, tiered designation system that concentrates resources in fewer institutions with higher specialization. Table 4 summarizes the institutional, patient, and workforce indicators.

### 4. Legal obligations for patient transport and acceptance

Transportation and acceptance of emergency patients constitute vital processes within EMS systems and play crucial roles in saving lives. This section compares the legal frameworks governing the transport and acceptance of emergency patients in Japan and South Korea with a focus on legal obligations and grounds for hospital refusal. While both countries are advanced Asian nations that have established sophisticated emergency care systems, major differences exist in their legal underpinnings.

Japan’s legal framework for emergency medicine has a multilayered structure. The Medical Care Act provides a statutory basis for designating emergency medical institutions through prefectural medical care planning [27], establishing a three-tier system of primary, secondary, and tertiary emergency care. However, operational governance of emergency patient transport and hospital acceptance is regulated separately: the Fire Service Act governs transport procedures [31], while the Medical Practitioners Act defines hospital acceptance obligations through the physician’s duty to respond to treatment requests [32]. This fragmented regulatory structure contrasts with Korea’s unified approach. This duality can result in less-than-seamless legal coordination between the transportation and acceptance phases of EMS care. South Korea's EMS crews operate under relevant legislation, namely the Act on 119 Rescue and Emergency Medical Services [33]. Provisions regarding medical directions by EMS physicians are also included in the act. However, the principles of patient admission and registration are comprehensively covered by the Emergency Medical Service Act in Korea [34].

Legal provisions regarding EMS patient acceptance also differ considerably between the two countries. In Japan, Article 35-5 of the Fire Service Act requires each prefecture to establish “standards for the transport and acceptance of the sick and injured” [35]. The obligation of medical institutions to accept patients is defined by Article 19 of the Medical Practitioners Act, which stipulates the so-called “duty of medical response.” This provision states that “a physician engaged in medical practice must not refuse a request for examination or treatment without just cause” [32]. The interpretation of “just cause” is broad, allowing refusal in cases such as limited treatment capacity, lack of necessary equipment, out-of-specialty cases, or when occupied with other patients.

In South Korea, Article 6 of the Emergency Medical Service Act sets forth a clear obligation to provide emergency medical care. Specifically, Article 6(1) requires emergency medical personnel at designated institutions to “faithfully perform their duties to be able to treat emergency patients at all times.” Article 6(2) stipulates that “emergency medical personnel shall immediately provide emergency care when requested or upon finding an emergency patient and must not refuse or evade such care without justifiable reason” [34]. While these provisions resemble Japan’s duty of medical response, the interpretation of “justifiable reason” in South Korea is much narrower, resulting in a substantially stricter obligation for medical institutions to accept EMS-transported patients. Japan’s Medical Practitioners Act, Article 19 (duty of medical response), does not provide for direct criminal penalties in the event of violation. However, severe violations may result in administrative sanctions such as revocation or suspension of a medical license under Article 7(1) of the same Act. However, in practice, administrative sanctions solely for breach of the duty of response are extremely rare [32]. In South Korea, Article 60(3) 1 of the Emergency Medical Service Act prescribes strict criminal penalties for those who violate Article 6(2) by refusing to provide or by evading emergency care. Offenders may face “imprisonment of up to three years or a fine of up to 30 million Korean won (21,600 USD)” [34]. In South Korea, strict penalties for refusal have helped reduce the so-called “ambulance ping-pong” phenomenon, although they may have also increased the burden on healthcare institutions [36]. In Japan, a higher degree of institutional autonomy leads to variations in EMS patient acceptance, depending on local medical resources [37]. Table 4 summarizes the legal frameworks in both countries.

### 5. Governance and quality assurance mechanisms

Both Japan and South Korea have established governance frameworks to ensure the quality and accountability of EMS systems. These frameworks encompass medical oversight systems, legal obligations, and institutional quality assurance mechanisms.

In Japan, medical directions are guided by local MC councils, ensuring physician oversight of all advanced interventions [19]. In South Korea, under the centralized governance structure of the NFA [18], emergency physicians are stationed at 119 Integrated Command Centers in certain regions to provide real-time medical oversight through radio or digital communication. The MOHW regularly evaluates designated emergency medical institutions to ensure compliance with national standards [23,29].

Although Japan does not impose a legal obligation on hospitals to accept EMS requests, operational mechanisms exist to manage situations in which multiple hospitals decline acceptance. In Japan, when multiple hospitals are unable to accept EMS-transported patients, ambulance crews follow a stepwise coordination process rather than a legally mandated acceptance requirement. Under the Fire Service Act, EMS personnel must continue contacting nearby hospitals based on proximity, patient condition, and availability of relevant specialties; however, hospitals retain broad discretion to decline acceptance because the Medical Practitioners Act does not impose a penalty-bearing duty to accept EMS requests [31,32]. When several hospitals refuse, ambulance crews receive support from MC physicians or regional medical information systems to identify an alternative facility with available capacity [35]. National surveys by the FDMA indicate that “difficulty in hospital acceptance” cases frequently require multiple inquiries, often five or more calls, before a receiving hospital is secured, particularly in metropolitan areas or during periods of high demand [37]. Although several prefectures operate regional hospital information systems that display bed or resource availability, these systems do not ensure acceptance [17]. This flexible, non-penal regulatory approach allows hospitals to manage workload and resource constraints but also contributes to regional variation and delays in securing destinations, in contrast to South Korea’s stronger statutory obligations for ED acceptance.

## Discussion

In this study, we conducted a comparative analysis of the structures, legal frameworks, specialist training systems, and real-world operations of EMS systems in Japan and South Korea, two advanced East Asian nations. Our findings revealed that while both countries face modern challenges such as population aging and disparities in regional healthcare resources, there are significant differences in legal underpinnings, institutional design, and frontline realities.

Regarding its legal framework, Japan features a dual system governed by the Fire Service Act and the Medical Practitioners Act, with separate regulations for the transport and acceptance of EMS patients. In contrast, South Korea has a dual system governed by the 119 Rescue and Emergency Medical Service Act. While both countries employ dual legal frameworks, South Korea’s unified operational implementation under the Emergency Medical Service Act enables more consistent coordination from prehospital transport to hospital acceptance of EMS patients. This difference influences the consistency and level of control in coordinating transportation and the acceptance of EMS patients. South Korea’s strict legal obligations and penalties may help prevent the so-called “ambulance ping-pong” phenomenon but may also increase the burden on healthcare professionals and reduce operational flexibility. Additionally, these stringent legal liabilities are causing specialists in various fields, including emergency medicine, to leave higher-level hospitals and constitute one of the factors discouraging physicians from applying to these specialties [30]. Additionally, in Korea, the barriers to accessing regional emergency medical centers as higher-level emergency medical centers are relatively low. Thus, the transport of emergency patients is often influenced not by the severity of the patient’s condition but by factors such as the distance from the incident location to the medical facility, the availability of medical resources at the time of the emergency, and the preferences of the patient or their guardian. These factors may contribute to less clarity in the functional distinctions between regional and local emergency centers. In Japan, a more flexible interpretation of the duty to accept patients and greater institutional autonomy may foster adaptability but can also lead to regional disparities and challenges with hospital acceptance of EMS transport.

Japan’s 4,100 dispersed institutions enable multilayered care with broad specialty participation and distribution of clinical workloads [22]. Conversely, South Korea’s 412 concentrated facilities have placed emergency physicians as universal entry points, creating higher individual workloads. These structural differences directly contribute to physician burnout and workforce shortages, which require consideration in future system designs.

Additionally, Japan’s “multi-disciplinary participation” model in emergency care allows a wide range of specialists to handle initial emergency care, with emergency physicians often focusing on severe cases, diagnostically challenging cases, or acting as coordinators of care. In contrast, South Korea’s system consistently places emergency medicine physicians at the front line for initial assessment, treatment, and triage of all emergency patients, emphasizing both concentrated responsibility and professional specialization. While this model ensures clear accountability and high standards, it may also lead to an excessive workload and an increased risk of delays or attrition when human resources are limited. These differences reflect not only institutional design but also medical culture, social expectations, hospital management policies, and career development pathways for physicians. Moving forward, the optimal allocation of medical resources, improvement of emergency physician work environments, and creation of flexible workforce models tailored to local needs are essential. It is clear from this study that both systems have their own strengths and limitations, and neither can be considered universally superior. Learning from the experiences and institutional practices of each system can contribute to the development of more efficient and humane EMS and emergency medicine frameworks. For example, Japan’s multilayered and decentralized model has implications for South Korea in terms of distributing emergency physician workloads and strengthening community healthcare resources.

South Korean patients prefer tertiary hospitals based on perceived quality [38], whereas Japanese patients prioritize proximity [39]. Both countries are experiencing substantial nonurgent ED visits, contributing to overcrowding and inefficient resource allocation. Recently, in South Korea, a mass exodus of residents and fellows from tertiary hospitals has created an acute shortage of available medical staff, leading to a reduction in the healthcare services available at these institutions [40]. A preliminary case series showed that total ED visits decreased by 25% to 40%, but the proportion of critical patients increased, intensifying the workload on the remaining medical personnel. A recent editorial in *The Lancet Regional Health - Western Pacific* warned that prolonged trainee shortages will further weaken already vulnerable services, such as emergency medicine, pediatrics, and obstetrics, and urged a swift policy compromise alongside broader workforce reforms [41]. Considering the findings of the present study, we believe that urgent and comprehensive improvements across the entire medical-social spectrum, including not only the workforce but also the legal framework and principles of remuneration, are needed to ensure the stable and sustainable provision of an optimal EMS as a social safety net.

This comparative study objectively clarified the features and differences between the Japanese and Korean emergency medical systems, presenting complementary lessons and pathways for future system improvements. The key policy messages include the following four items: First, regulatory frameworks must balance enforcement with provider sustainability; second, public education regarding appropriate EMS utilization is crucial for system efficiency; third, both decentralized and centralized models offer distinct advantages for the development of hybrid approaches; and fourth, enhanced cross-national collaboration through shared research and training can strengthen regional EMS and emergency medicine resilience.

## Figures and Tables

**Table 1.** Comparison of emergency medicine specialist frameworks in Japan and South Korea

Category	Japan	South Korea	Data year
Year of establishment/society name	1973/JAAM [5]	1989/KSEM [6]	Historical
Founding Background	Developed in response to increased traffic and industrial trauma, aiming to enhance trauma care systems [5]	Modeled after the North American EM system, aiming to train comprehensive emergency physicians [7]	Historical
Number of certified emergency physicians	6,139 (as of January 2025) [8]	2,464 (as of 2024) [11]	January 2025/2024
	Approximately 5.7% of all board-certified specialists	Approximately 2.5% of all board-certified specialists	
Specialists per 100,000 population	4.96 [8,10]	4.81 [11,12]	2024–2025
Subspecialty system in emergency medicine	Managed by related academic societies other than JAAM [8]	Structured programs managed by KSEM and affiliated societies [7,14]	2024–2025
Common subspecialties in emergency medicine	Intensive care medicine, clinical toxicology, pediatric emergency medicine, etc. [8]	Toxicology, trauma surgery, critical care, pediatric emergency medicine, disaster and prehospital medicine [7]	2024

JAAM, Japanese Association for Acute Medicine; KSEM, Korean Society of Emergency Medicine; EM, emergency medicine.

**Table 2.** Summary comparison of emergency medical service systems in Japan and South Korea

Category	Japan	South Korea	Data year
Administrative model	Decentralized (municipality-based) [16]	Centralized (national governance) [18]	2024
Fire department count	722 fire departments [16,17]	1 National + 19 regional HQs (~250 stations) [18]	2024
Emergency response teams	5,415 teams [17]	Data not specified	April 2024
Ambulances	6,640 (including reserve vehicles) [17]	1,881 (including reserve vehicles) [21]	April 2024/December 2024
Ambulances staffing	3 crew members, including ≥1 ELST [19]	2–3 crew, including ≥1 Level 1 EMT or Registered Nurse [20,21]	2024
Total emergency medical personnel	67,006 [17]	14,236 [21]	April 2024/December 2024
Certified advanced paramedics	33,350 ELSTs (49.8% of personnel) [17]	5,347 Level 1 EMTs (37.6% of personnel) [21]	April 2024/December 2024
Other certified personnel	33,656 EMTs (50.2% of personnel) [17]	2,253 Level 2 EMTs; 4,290 Registered Nurses [21]	April 2024/December 2024
Command centers	Municipal command centers, regionalization in progress [16,17]	Integrated 119 centers with real-time IT systems [18]	2024
Teams with advanced provider	99.6% of teams have ≥1 ELST [17]	Data not specified	April 2024

HQ, headquarter; ELST, emergency life-saving technician; EMT, emergency medical technician; IT, information technology.

**Table 3.** Comparison of emergency care systems and institutional capacity in Japan and South Korea

Category	Japan	South Korea	Data year
Emergency care structure			
System type	Three-tier system: Primary–Secondary–Tertiary [22]	Tiered classification by institutional capacity (3 types) [23,24]	2024
Allocation criteria	Clear division by patient severity [22]	Based on facility function, region, and case severity [23,24]	2024
Facilities for severe cases	Critical Care Medical Centers [27]	Regional Emergency Medical Centers, Trauma Centers [29]	2024
Transport coordination	Protocol-based triage and MC physician-guided hospital selection [22]	Field crew judgment and patient preference [25,26]	2024
National regulation	Nationwide standardization under legal framework [16]	National designation system with regional implementation [18]	2024
Institutional capacity			
Total emergency institutions	Approximately 4,100 [27]	412 designated facilities [29]	2022–2024
Tertiary/regional centers	~300 Critical Care Medical Centers [27]	42 Regional Emergency Medical Centers [29]	2024
Operational volume			
Annual ambulance transports	6.64 million (2023) [17]	~3.5 million (2023) [29]	2023
Critical cases (death+severe)	8.6% (1.3% death, 7.3% severe) [17]	~5%–10% [30]	2023

MC, Medical Control.

**Table 4.** Comparison of legal framework for transport and acceptance of emergency patients in Japan and South Korea

Category	Japan	South Korea
Legal framework	Multilayered structure: Medical Care Act (for facility designation and medical care planning) [27]; Fire Service Act (for transport) [31]; Medical Practitioners Act (for acceptance) [32]	Dual structure: Act on 119 Rescue and Emergency Medical Services (for 119 organization and medical direction) [33] and Emergency Medical Service Act (comprehensive medical regulation) [34]
Post-transport acceptance	“Standards for the transport and acceptance of the sick and injured” set by each prefecture under Article 35-5 [31,35]; not binding on hospitals	Article 6(2) of the Emergency Medical Service Act [34]: refusal or evasion of emergency care prohibited except for justifiable reasons
Physician’s ethical duty	Article 19 of the Medical Practitioners Act [32]: duty of response; refusal not permitted without just cause	Article 6(1) of the Emergency Medical Service Act [34]: duty to always be able to treat emergency patients diligently
Grounds for EMS refusal	Refusal allowed if there is just cause (e.g., limited capacity, lack of equipment, full beds, attending other patients) [31,32]	Refusal not permitted unless there is a justifiable reason; only specifically defined reasons (e.g., violence against staff) are accepted [34]
Penalties for EMS refusal	No direct penalties for breach of duty of response; possible administrative sanctions, but no criminal penalties [31,32]	Article 60(3) 1 [34]: criminal penalties for violation of Article 6(2) (up to 3 years imprisonment or 30 million Korean won fine)
Coordination mechanism	Stepwise hospital contact process supported by MC physicians and regional medical information systems [19]; hospitals often require 5 or more inquiries in difficult cases [17,35]	Integrated 119 system with real-time hospital capacity monitoring [18]; penalties for refusal reduce “ambulance ping-pong” phenomenon [36]
Regional variation	Significant regional variation in hospital acceptance rates and transport times [17,35]; metropolitan areas face higher rates of “difficult-to-transport” cases	More standardized acceptance process due to centralized governance and legal penalties [18,34]; regional disparities remain in rural areas

EMS, emergency medical services; MC, Medical Control.

## Supplementary Materials

Supplementary Tables 1, 2, and Supplementary Fig. 1 can be found at https://doi.org/10.12701/jyms.2026.43.3.

Supplementary Table 1. Search strategy by database

Supplementary Table 2. Inclusion and exclusion criteria

Supplementary Figure 1. Document Selection Process.
