# Supplementary material for: Comparing emergency medical system governance in Japan and South Korea: lessons for high-income countries from a multisource comparative health systems analysis
Source: J Yeungnam Med Sci. 2025 Dec 18;43:3. doi: 10.12701/jyms.2026.43.3 (PMC12887121; doi:10.12701/jyms.2026.43.3)
Supplement: Supplementary Table 1. — Search strategy by database [file jyms-2026-43-3-Supplementary-Table-1.pdf]

Supplementary Table 1. Search strategy by database

Database	Search term
PubMed	("emergency medical services"[MeSH] OR "EMS"[tiab]) AND ("Japan"[tiab] OR "South Korea"[tiab] OR "Korea"[tiab]) AND ("governance" OR "system" OR "medical control" OR "law")
Scopus	TITLE-ABS-KEY("emergency medical services" OR "EMS") AND ("Japan" OR "South Korea") AND ("governance" OR "system structure" OR "medical control")
CiNii / J-STAGE	"救急医療" AND ("体制" OR "システム" OR "制度" OR "メディカルコントロール" OR "医師法" OR "消防法" OR "救急救命士 " OR "医療法"OR "専門医")
KISS	"응급의료" AND ("체계" OR "시스템" OR "메디컬컨트롤" OR "응급의료법" OR "전문의" OR "이송기준")
KoreaMed	("emergency medical service" OR "EMS") AND ("Korea") AND ("system" OR "law" OR "governance")
RISS	"응급의료체계" OR "119구급" AND ("법률" OR "제도" OR "전문의")

Search strategies used for document identification. All searches covered January 2000 to July 2025. Documents were selected based on relevance to five thematic domains rather than exhaustive retrieval.
Key Japanese terms: 救急医療 (emergency care), 体制/システム (system), 制度 (institution/framework), メディカルコントロール (medical control), 医師法 (Medical Practitioners Act), 消防法 (Fire Service Act), 救急救命士法 (Paramedics Act), 医療法 (Medical Care Act),専門医 (board-certified specialist).
Key Korean terms: 응급의료 (emergency care), 체계/시스템 (system), 메디컬컨트롤 (medical control), 응급의료법 (Emergency Medical Service Act), 전문의 (specialist), 이송기준 (transport criteria).
Additional sources: Government websites (Fire and Disaster Management Agency [FDMA] of Japan; National Fire Agency [NFA] of South Korea; Ministry of Health, Labour and Welfare [MHLW] of Japan; Ministry of Health and Welfare [MOHW] of South Korea), legal databases (e-Gov Laws and Regulations Search [Japan]; Korea Legislation Research Institute [KLRI]), professional society reports (Japanese Association for Acute Medicine [JAAM]; Korean Society of Emergency Medicine [KSEM]), reference list screening, and expert input.
