# Supplementary material for: Comparing emergency medical system governance in Japan and South Korea: lessons for high-income countries from a multisource comparative health systems analysis
Source: J Yeungnam Med Sci. 2025 Dec 18;43:3. doi: 10.12701/jyms.2026.43.3 (PMC12887121; doi:10.12701/jyms.2026.43.3)
Supplement: Supplementary Table 2. — Inclusion and exclusion criteria [file jyms-2026-43-3-Supplementary-Table-2.pdf]

Supplementary Table 2. Inclusion and exclusion criteria

Criteria	Detail
Inclusion criteria	
Time period	January 2000–July 2025
Languages	English, Japanese, Korean
Content focus	EMS structure, governance, workforce, legal frameworks, operational data relevant to comparative analysis
Document types	Peer-reviewed articles, government reports, white papers, legal documents, official statistics, professional society publications
Exclusion criteria	
Content scope	Clinical outcome studies without system-level structural or organizational analysis
Language	Publications in languages other than English, Japanese, or Korean
Quality	Opinion pieces or editorials without empirical data; documents lacking verifiable system-level information

Thematic domains: Documents were selected based on relevance to five thematic domains: (1) specialist training and certification, (2) prehospital EMS organization, (3) hospital-based emergency care structure, (4) legal obligations for patient transport and acceptance, and (5) governance and quality assurance mechanisms. Priority was given to the most recent and authoritative sources for each domain.
