# Supplementary material for: Comparing emergency medical system governance in Japan and South Korea: lessons for high-income countries from a multisource comparative health systems analysis
Source: J Yeungnam Med Sci. 2025 Dec 18;43:3. doi: 10.12701/jyms.2026.43.3 (PMC12887121; doi:10.12701/jyms.2026.43.3)
Supplement: Supplementary Figure 1. — Document Selection Process. [file jyms-2026-43-3-Supplementary-Fig-1.pdf]

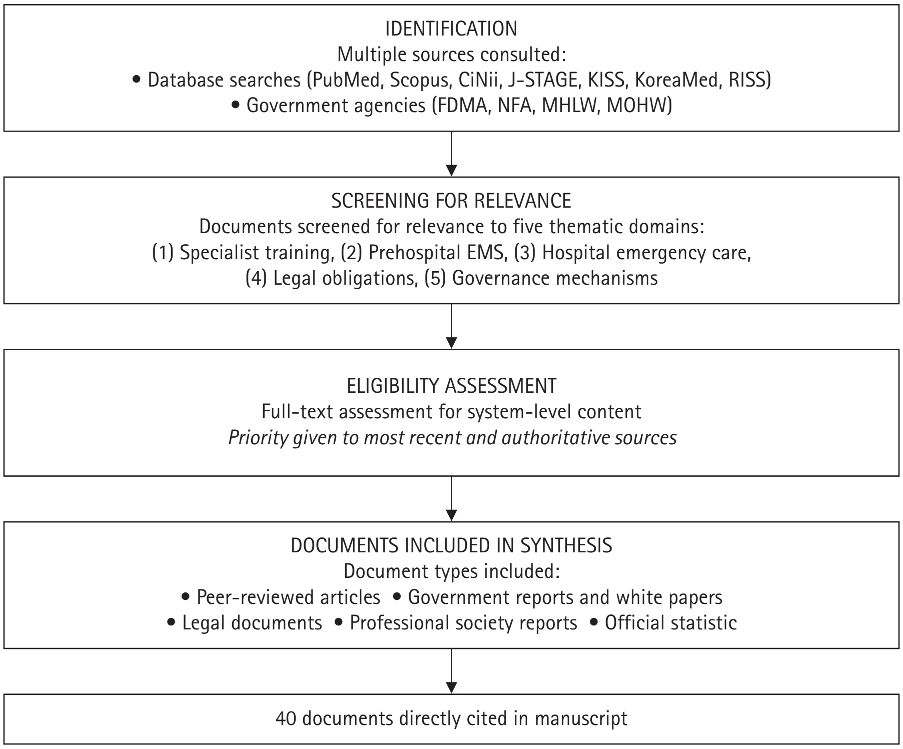

Supplementary Fig. 1. Document selection process.
Flowchart illustrating the document identification, screening, and selection process for this targeted literature review comparing emergency medical services governance in Japan and South Korea.
